# Automated laryngeal mass detection algorithm for home-based self-screening test based on convolutional neural network

**DOI:** 10.1186/s12938-021-00886-4

**Published:** 2021-05-25

**Authors:** Gun Ho Kim, Eui-Suk Sung, Kyoung Won Nam

**Affiliations:** 1 Interdisciplinary Program in Biomedical Engineering, School of Medicine, Pusan National University, Busan, South Korea; 2 Department of Otolaryngology-Head and Neck Surgery, Pusan National University Yangsan Hospital, Yangsan, South Korea; 3 Research Institute for Convergence of Biomedical Science and Technology, Pusan National University Yangsan Hospital, Yangsan, South Korea; 4 Department of Biomedical Engineering, Pusan National University Yangsan Hospital, Yangsan, South Korea; 5 Department of Biomedical Engineering, School of Medicine, Pusan National University, 49 Busandaehak-ro, Mulgeum-eup, Yangsan, Gyeongsangnam-do 50629 South Korea

**Keywords:** Laryngeal mass, Convolutional neural network, Deep learning, Patient safety

## Abstract

**Background:**

Early detection of laryngeal masses without periodic visits to hospitals is essential for improving the possibility of full recovery and the long-term survival ratio after prompt treatment, as well as reducing the risk of clinical infection.

**Results:**

We first propose a convolutional neural network model for automated laryngeal mass detection based on diagnostic images captured at hospitals. Thereafter, we propose a pilot system, composed of an embedded controller, a camera module, and an LCD display, that can be utilized for a home-based self-screening test. In terms of evaluating the model’s performance, the experimental results indicated a final validation loss of 0.9152 and a F1-score of 0.8371 before post-processing. Additionally, the F1-score of the original computer algorithm with respect to 100 randomly selected color-printed test images was 0.8534 after post-processing while that of the embedded pilot system was 0.7672.

**Conclusions:**

The proposed technique is expected to increase the ratio of early detection of laryngeal masses without the risk of clinical infection spread, which could help improve convenience and ensure safety of individuals, patients, and medical staff.

## Background

The vocal cord vibrates to produce voice. Masses such as nodules, polyps, granulomas, or tumors near the vocal cord can induce various clinical symptoms such as hoarseness, breathiness, abnormal voice, pain in the ear or neck, and even laryngeal cancer [1]. Several recent studies have reported the clinical effects of laryngeal masses, such as airway obstruction, tracheostomy, and reflux diseases [2–5]. When an individual visits a hospital because of a voice disorder or pain, an otolaryngologist first examines their throat using a laryngoscope to check for any structural abnormalities or color changes on or near the vocal cord and larynx. If a mass is found on or near the vocal cord during the endoscopic diagnosis, further examinations such as additional imaging or pathological diagnosis are performed to identify the type and severity of the disease, that is, to verify whether the mass is benign (needs periodic observation) or malignant (needs microlaryngoscopic surgery).

Similar to other masses in various body parts that can worsen to cancers over time, it is essential to detect the generation of a laryngeal mass on or near the vocal cord early to improve the possibility of full recovery and the long-term survival ratio after medication treatment or surgery. However, unlike masses on the skin that are easy to identify at home using the naked eye, masses on or near the vocal cord are not easily observable at home for most individuals. Therefore, a healthy individual who would like to check the status around their vocal cord for preventive purposes would have to periodically visit a hospital for endoscopic diagnosis, which may cause inconveniences. Recently, the psychological repulsion to visit crowded hospitals for preventive inspection without any self-observable symptoms has been increasing owing to the spread of highly dangerous infectious diseases, such as COVID-19. Furthermore, there is a risk of clinical transmission of infectious diseases by unconscious virus carriers who do not require emergency medical treatment. To detect the generation of laryngeal masses early while preventing the risk of transmission of clinical infection, a reliable and easy-to-use technical tool for home-based self-screening inspection of laryngeal masses is required.

In this paper, we first propose a convolutional neural network (CNN)-based artificial intelligence (AI) model for automated laryngeal mass detection. Thereafter, we propose a pilot system, composed of an embedded controller, a camera module, and an LCD display, for a home-based self-screening test.

## Results

Table 1 summarizes the performance of the implemented Mask RCNN model for various augmentation strategies and confidence levels before additional post-processing. Of the total number of overall augmentation–confidence level combinations evaluated, the combination of a single augmentation–80% confidence level yielded maximal accuracy (0.7322) and F1-score (0.8371) for mass detection among the tested conditions.

**Table 1:** Comparison of vocal cord frame detection results using two augmentation strategies and three confidence levels

Target	Aug	Conf (%)	TP	TN	FN	FP	Rec	Pre	Acc	F1-score
Vocal cord	No-aug	80	231	0	11	0	0.9545	1.0000	0.9545	0.9767
		85	237	0	5	1	0.9793	0.9958	0.9753	0.9875
		90	229	0	13	0	0.9502	1.0000	0.9502	0.9745
	Single	80	236	0	6	1	0.9752	0.9958	0.9712	0.9854
		85	230	0	11	2	0.9544	0.9914	0.9465	0.9725
		90	238	0	4	1	0.9835	0.9958	0.794	0.9896
	Mixed	80	235	0	7	4	0.9711	0.9833	0.9553	0.9771
		85	236	0	6	4	0.9752	0.9833	0.9594	0.9793
		90	232	0	10	3	0.9587	0.9872	0.9469	0.9727
Mass	No-aug	80	153	13	96	36	0.6145	0.8095	0.5570	0.6986
		85	140	14	106	27	0.5691	0.8383	0.5366	0.6780
		90	161	14	85	31	0.6545	0.8385	0.6014	0.7352
	Single	80	203	13	45	34	0.8185	0.8565	0.7322	0.8371
		85	207	14	43	48	0.8280	0.8118	0.7083	0.8198
		90	175	13	73	26	0.7056	0.8706	0.6551	0.7795
	Mixed	80	200	12	48	34	0.8065	0.8547	0.7211	0.8299
		85	210	13	43	46	0.8300	0.8203	0.7147	0.8251
		90	193	14	55	39	0.7782	0.8319	0.6877	0.8042

*Aug* augmentation, *Conf* confidence level, *TP* true-positive, *FP* false-positive, *TN* true-negative, *FN* false-negative, *Rec* recall, *Pre* precision, *Acc* accuracy

**Fig. 1:**
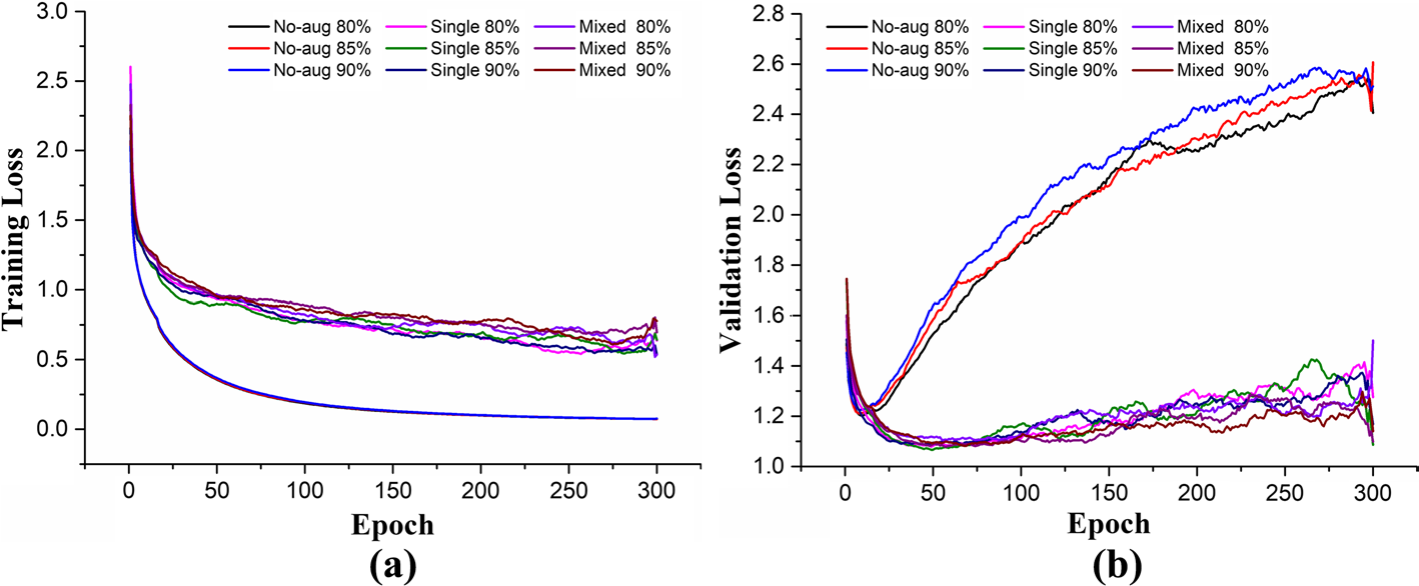
Variations in the training and validation losses of the implemented Mask RCNN model for increasing epochs up to 300. The original graphs were smoothed using an adjacent averaging filter (*n* = 30) to improve comprehension **a** training loss and **b** validation loss

Figure 1 presents the variations in the training/validation losses when the epoch value increases from 1 to 300. The validation loss was minimized (0.9152) when the epoch value was 260 in the [single augmentation–80% confidence level] condition, which exhibited the highest F1-score in Table 1.

**Fig. 2:**
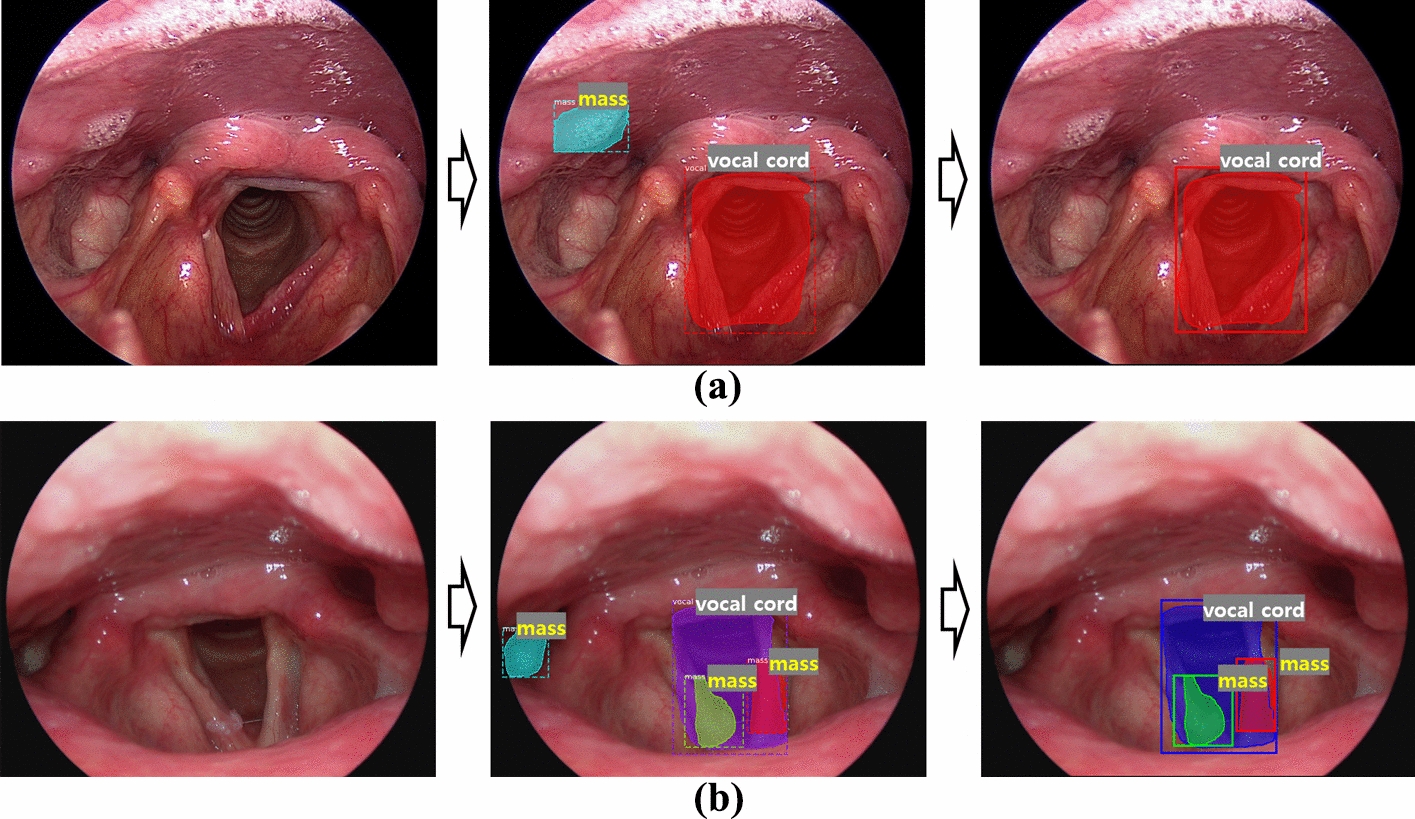
Two examples of ROI extraction for vocal cord and mass, presented in the following order: original image (left), ROIs from implemented Mask RCNN model (center), and ROIs after post-processing (right): **a** image for mass-included case and **b** image for no-mass case. *ROI* region-of-interest

Figure 2 demonstrates the effect of excluding the false mass cases using the post-processing technique described in the methods section when the [single augmentation–80% confidence level] combination was applied in the implemented Mask RCNN model. The false cases were successfully excluded from the final prescreening output. Table 2 lists the quantitative performance parameters of the implemented prescreening algorithm before and after applying post-processing. The number of false-positive cases decreased from 34 to 26, and the F1-score increased from 0.8371 to 0.8534 after post-processing was applied as desired. Additionally, the number of false-negative cases reduced from 45 to 41.

**Table 2:** Mass detection results before/after post-processing to reduce the number of false-positive cases for the [single augmentation–80% confidence level] condition

Post-processing	TP	TN	FN	FP	Rec	Pre	Acc	F1-score
Before	203	13	45	34	0.8185	0.8565	0.7322	0.8371
After	195	13	41	26	0.8263	0.8824	0.7564	0.8534

When evaluating the performance of the embedded pilot system for the home-based self-screening test, the pilot system could successfully detect the vocal cords in 99 images (*TP* = 99, *TN* = 0, *FN* = 1, *FP* = 0; recall = 0.99, precision = 1.00, accuracy = 0.99, F1-score = 0.99). Furthermore, the system successfully detected 89 masses, did not detect 22 masses, and misdetected 32 clean tissues as masses (*TP* = 89, *TN* = 0, *FN* = 22, *FP* = 32, recall = 0.8018, precision = 0.7355, accuracy = 0.62, F1-score = 0.7672). When comparing these results with those from the original computer algorithm, the F1-score of the pilot system (0.7672) was lower than that of the original computer algorithm (0.8534). Additionally, the running time of the model was approximately 40 s on the embedded pilot system, whereas it was within 5 s on the computer.

## Discussion

For image augmentation, four types of augmentation strategies—flip, rotation, addition, and affine—were selected considering realistic diagnosis circumstances as follows. First, in actual circumstances, patients have masses either on the left side, the right side, or both sides of their vocal cords. To reflect this positional variation while training the model, a horizontal flip option was included. Second, the vertical flip and rotation options were included to reflect variations in the handgrasping position of the handle and the entry angle and orientation of the endoscopic camera during diagnosis. Third, the hardware characteristics of commercial camera modules, such as image brightness, resolution, and RGB color characteristics, are somewhat different from each other. Therefore, the addition option was included to reflect the variation in the RGB characteristics of the camera module. Fourth, the affine option was included to reflect the variation in the distance between the camera and vocal cord during diagnosis.

Most previously reported endoscopic mass detection studies have targeted colon polyps during screening for colon cancer. Before the era of AI, which can be represented by deep learning, researchers adopted manual or semi-automatic colon polyp detection methodologies using handcrafted features that were determined by human researchers. For example, Tajbakhsh et al. [6] pre-processed colonoscopy images using their unique feature extraction and edge classification schemes and utilized context and shape information to localize polyps. Silva et al. [7] extracted possible polyps within wireless capsule endoscopic images using geometric shape features and evaluated candidate regions using a boosting-based method with textural features. Recently, owing to the rapid advances in AI technologies, researchers have reported several fully automatic colon polyp detection studies that applied colonoscopy images to a deep learning model [8–12]. In the case of laryngeal masses, several studies using handcrafted features have been reported. For example, Wang et al. [13] proposed a method for throat polyp detection based on singular value decomposition and support vector machines using vowel voices of patients. Turkmen et al. [14] proposed a machine learning algorithm that classifies laryngeal disorders into healthy, nodule, polyp, laryngitis, and sulcus vocalis. To the best of our knowledge, deep learning techniques have not been applied so far for the fully automatic detection of laryngeal masses, which forms the novelty of our study. Additionally, in Wang’s study [13], the maximal correct rate of prediction was approximately 0.9, whereas in Turkmen’s study [14], the sensitivity of polyp detection was approximately 80%. In our study, the value of recall for laryngeal mass detection was 0.8263 in the [single augmentation–80% confidence level] condition, showing an almost equivalent performance compared to results reported in previous studies. Moreover, previous studies could only estimate the possible existence of laryngeal polyps. In contrast, the proposed method can also indicate the suspected regions of the laryngeal mass from each image, which is more suitable for home-based self-screening purposes for non-experts.

Most previous studies have focused only on evaluating the performance of their own deep learning models using clinical diagnostic data; they have not attempted to expand their model to home-based self-screening. However, with the spread of COVID-19, the risk of clinical infection spreading from medical staff to patients or from one patient to others has increased. Therefore, to avoid unnecessary repetitive hospital visits for non-serious patients or healthy individuals, the significance of contactless medical diagnosis and reliable self-screening at home has promptly increased. In this paper, we propose a pilot system for automated laryngeal mass detection that can be utilized as a tool for home-based self-screening. The experimental results indicated that the pilot system performed reasonably well (F1 score = 0.7672) for home-based self-screening considering the additional artifacts during the photographing, such as the characteristics of the color printer, effects of the environmental lights, and subtle vibration of the hand, which implied the possibility of home-based self-diagnosis of laryngeal masses using an inexpensive, portable, and easy-to-use embedded device. Using this self-diagnostic tool, it is possible to detect early laryngeal mass generation remotely without having to visit the hospital, which can improve convenience and ensure the safety of individuals by reducing the risk of clinical infection spread.

The proposed technique for automated laryngeal mass detection can be extended to various healthcare and medical applications. For example, we utilized Raspberry Pi as an embedded controller in the current study to ease implementation by Python code sharing. However, if the proposed AI model is ported to operate on a smartphone platform, user accessibility and convenience can be improved; all an individual needs to do is buy a commercial endoscope camera and download a phone app from the app library. If necessary, contactless counseling from a doctor can also be made available by sharing the photographed image through a cloud counseling platform. Second, if the proposed AI model is modified to be able to track laryngeal masses in real time for streaming images, it can help doctors during clinical examination. For example, an AI-supporting device, such as a laptop, can be connected to the video-out port of an endoscope through a cable to obtain a livestream of the endoscopic images with the real-time results of laryngeal mass tracking displayed on the screen.

However, this study has certain limitations. First, during the evaluation of the model, the vocal cord was not detected in six images because of excessive deformation, and as a result, eight true-positives were also not detected. When such errors occur in an actual home-based self-screening situation, the individual can discard the result, adjust the position and angle of the camera tip, and re-perform the self-screening test to obtain appropriate screening results. Therefore, we identified such cases as non-serious errors (considered outliers) and excluded them from the statistical analysis. Second, because we received approval from the Institutional Review Board (IRB) for a retrospective study using diagnostic images from the PACS database rather than approval for performing actual subject tests using an endoscopic camera, we utilized color-printed images of the laryngeal mass and a conventional web camera to monitor the performance of the implemented pilot system. It may be necessary to perform further clinical trials in actual self-screening situations with fresh IRB approval to verify the clinical feasibility of the implemented pilot system.

## Conclusions

In this study, a CNN-based automated laryngeal mass detection algorithm and an embedded pilot system for home-based self-screening were proposed. The experimental results indicated the performance and feasibility of these implementations as tools for home-based self-screening purposes. The proposed technique is expected to increase the early detection of laryngeal masses without the risk of clinical infection spread, which improves convenience and ensures the safety of individuals, patients, and medical staff.

## Methods

### Image preparation for model training and validation

This retrospective study was approved by the IRB of the Pusan National University Yangsan Hospital (No. 05-2019-008) with the full cooperation of the Department of Otolaryngology-Head and Neck Surgery. To acquire the diagnostic images for model training, validation, and evaluation, we selected 1224 original images from the PACS database through full visual inspection by a qualified doctor (1153 images for mass-included cases [GRP_M] and 71 images for no-mass cases [GRP_C]). To acquire anonymous (no personal information in the image) DICOM images from the PACS database, we used a de-identification option of the system while saving the JPG files for the retrospective study. The acquired images were randomly divided into three groups: training, validation, and test datasets. The ratio of images in the training, validation, and test datasets was set to 3:1:1 (693 images from GRP_M and 43 images from GRP_C for training, 232 images from GRP_M and 14 images from GRP_C for validation, and 228 images from GRP_M and 14 images from GRP_C for evaluation). Thereafter, the positions of the target area (vocal cord and mass for GRP_M and vocal cord for GRP_C) in each image were manually marked by a trained expert. The annotation process for the detection target was performed using a popular web-based software (VGG Image Annotator (VIA) Version 2.0.9; Visual Geometry Group, Oxford, UK) [15]. The condition for the annotation was set to “polygon” and the results of the annotation for the overall original images were stored in a single JSON file.

### Model implementation for automated laryngeal mass detection

**Fig. 3:**
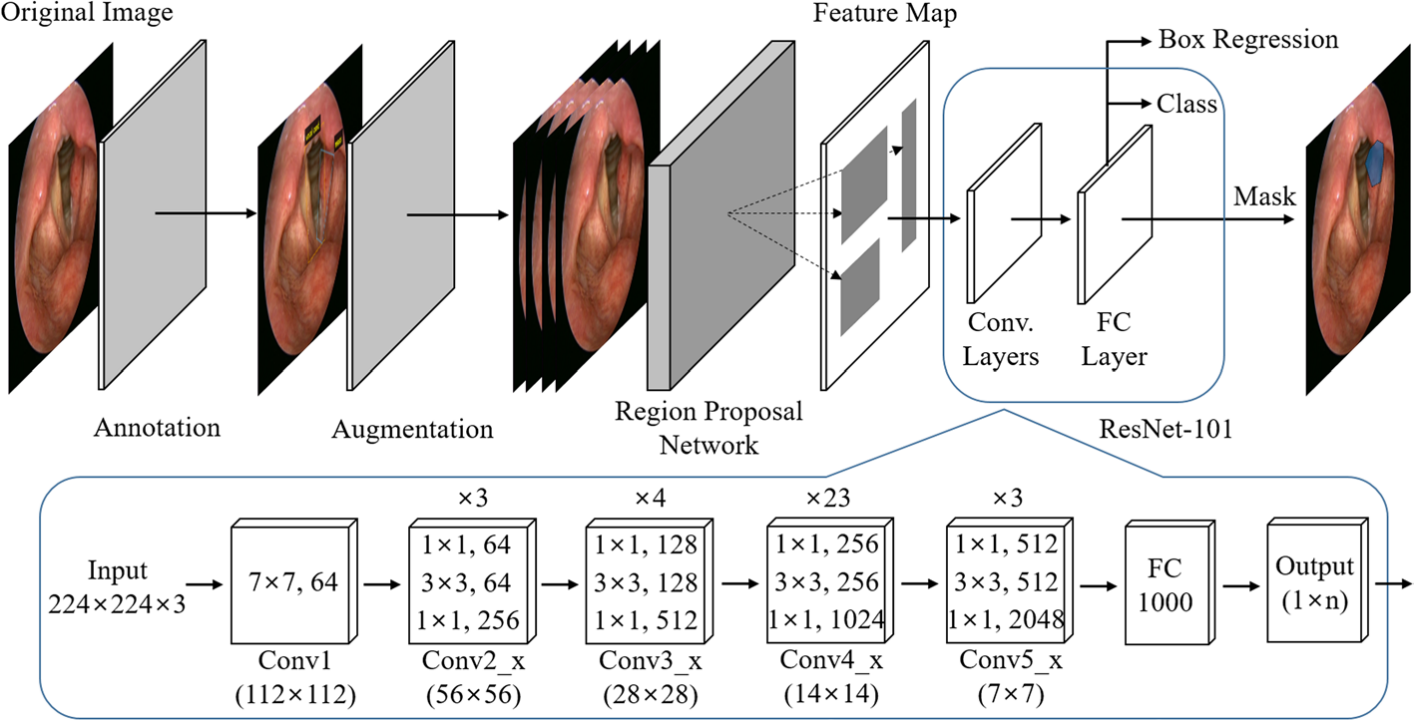
Structure of the proposed algorithm to detect vocal cords and masses near vocal cords from laryngeal endoscopic images. *FC* fully connected, *Conv*. convolution

We utilized an NVIDIA Geforce RTX2060 board, Anaconda 3.7, Python 3.6.10, Tensorflow 1.13, Keras 2.0.8, and CUDA 10.0 on Ubuntu 18.04.4 LTS for model development. Figure 3 shows the structure of the implemented CNN-based laryngeal mass detection algorithm.

**Fig. 4:**
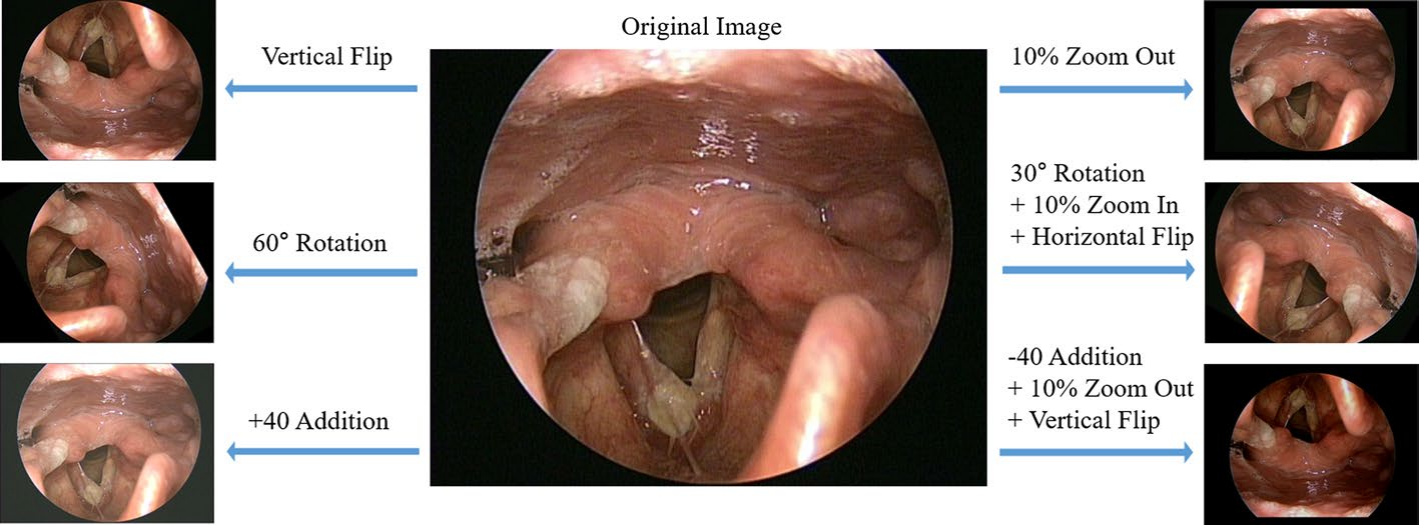
Example of image augmentation for each original image

When an original image and its annotation information are input to the algorithm, the image is first augmented using a popular image augmentation library (Imgaug, ver. 0.4.0) [16]. Considering the actual diagnosis circumstances, we applied five augmentation options to each original image: vertical flip, horizontal flip, rotation (0°–330° at intervals of 30°), addition (+ 40/− 40), and affine (10% zoom in/out) (Fig. 4). Thereafter, the augmented images were transmitted to the input layer of the object-detection network. We utilized a Mask RCNN model (Matterport; MIT) with ResNet-101, which was downloaded from GitHub as a backbone [17, 18]. Next, to improve the accuracy of target detection using the Mask RCNN model by reducing the possibility of model bias owing to the imbalance of image numbers between GRP_C and GRP_M in the private dataset, we downloaded COCO pre-trained weights for the Mask RCNN model from GitHub and applied the downloaded weights to the initial model. Subsequently, the model was trained using a private training dataset acquired from the PACS database. During transfer learning, we trained the model for 300 epochs using stochastic gradient descent with 100 training steps per epoch, a momentum of 0.9, and a learning rate of 0.001 by considering the general values of hyper-parameters in several previous works. We used a batch size of two on a single graphics processing unit.

During the model-based prescreening test, two types of errors can occur: (1) a clean tissue may be mistaken for a mass (false-negative), and (2) a mass may be mistaken as a clean tissue (false-positive). In the former case, an individual may go to the hospital and request a doctor to perform a secondary manual inspection of the suspected masses; thus, when an actual mass exists, it may be considered lightly. However, in the latter case, an individual who trusts the prescreening application may not go to the hospital, thus preventing early detection of the mass and inducing malignant laryngeal tumors. Because of this asymmetric risk, it is necessary to reduce the occurrence of false-positive cases and simultaneously improve the sensitivity of the algorithm.

During the evaluation of the implemented Mask RCNN model, clear tissues located far from the vocal cord with image characteristics similar to masses were mistaken as mass candidates in several test images, which led to an increased number of false positives (see Fig. 2). Further, the primary purpose of the proposed prescreening application was to detect mass candidates located near the vocal cords. Therefore, to exclude false positives from the final prescreening results, additional post-processing was performed on the output of the implemented Mask RCNN model as follows. First, suspect cases of the vocal cord and masses in the test image were extracted using the trained model under the [single augmentation–85% confidence level] condition (see Table 1). Second, the two-dimensional coordinates of the upper left and lower right corners were extracted from each of the rectangular suspect areas. Finally, suspected masses whose areas did not overlap with those of the vocal cord candidates were excluded from the list of suspected samples.

### Implementation of pilot system for home-based self-screening test

To implement the embedded controller-based pilot system for the home-based self-screening of laryngeal masses using the CNN model described above, a commercial embedded board (Raspberry Pi 4B; Raspberry Pi Foundation, Cambridge, UK; 4G RAM) was selected as a platform and Raspberry Pi OS with Python 3.7, Tensorflow 1.13.1, and Keras 2.0.8 were installed in a virtual environment. Thereafter, a camera module (C922 Pro Stream™; Logitech International S.A., Lausanne, Switzerland; 1920 × 1080 resolution, 30 frames/s), an LCD panel (Raspberry Pi3 Touchscreen Display; OKdo Technology Ltd., London, UK; 800 × 480 pixels, 7 in.), and a mouse were attached to the embedded board. Subsequently, a well-trained CNN model was ported to operate on the pilot system.

### Performance evaluation

To verify the effects of the selection of the minimum detection level within the Mask RCNN model and the setting of the augmentation library on the performance of the implemented algorithm, we adjusted the minimum detection levels to 80%, 85%, and 90%. We further adjusted the augmentation options as no-augmentation, single (i.e., a randomly selected augmentation is applied to the original image per epoch), and mixed (i.e., three randomly selected augmentations are simultaneously applied to the original image per epoch).

**Fig. 5:**
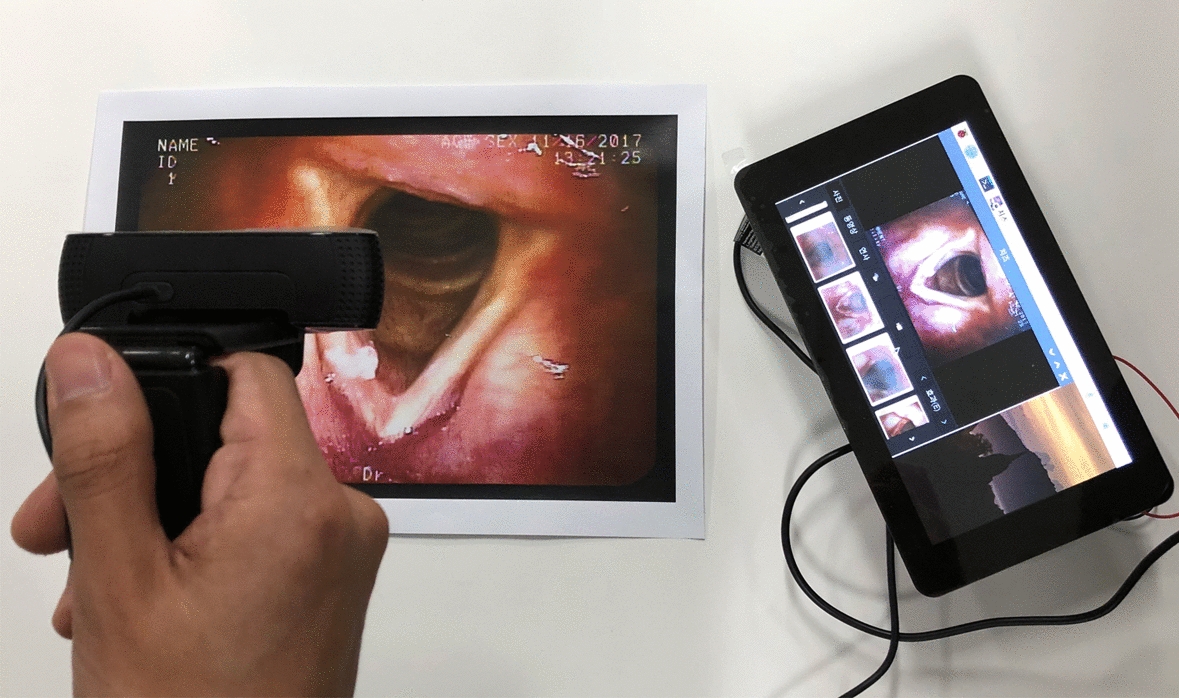
Performance evaluation of pilot system implemented for home-based self-screening test using 100 color-printed images

To verify the performance of the implemented pilot system for the home-based self-screening test, 100 mass-included original images (GRP_M) were randomly selected from the evaluation dataset. Each selected image was color-printed for testing, and each image was photographed using the pilot system. Subsequently, the results of laryngeal mass detection obtained from the pilot system were compared with those obtained using the original computer algorithm (Fig. 5).

## Data Availability

Not applicable.

## Abbreviations

CNN: Convolutional neural network
AI: Artificial intelligence
ROI: Region of interest
GRP_M: Images for mass-included cases
GRP_C: Images for no-mass cases
TP: True positive
TN: True negative
FP: False positive
FN: False negative
